# Association between plasma metal element profiles and cognitive impairment in occupationally aluminum-exposed workers at a large aluminum plant in northern China

**DOI:** 10.1016/j.tjpad.2025.100470

**Published:** 2026-01-01

**Authors:** Xin Guo, Fangyu Gao, Mujia Li, Baolong Pan, Feng Gao, Shanshan Wang, Jingsi Zhang, Xiaoting Lu, Jing Song, Linping Wang, Huifang Zhang, Qiao Niu

**Affiliations:** a Section of Occupational Medicine, Department of Special Medicine, Shanxi Medical University, Taiyuan, Shanxi 030001, China; b Department of Occupational Health, School of Public Health, Shanxi Key Laboratory of Environmental Health Impairment and Prevention, NHC Key Laboratory of Pneumoconiosis, MOE Key Laboratory of Coal Environmental Pathogenicity and Prevention, Shanxi Medical University, Taiyuan 030001, China; c Sixth Hospital of Shanxi Medical University, Taiyuan, Shanxi 030008, China

**Keywords:** Occupational aluminum exposure, Cognitive function, Plasma metal elements, Bayesian kernel regression, Occupational health

## Abstract

This study explored the association between plasma levels of multiple metals and cognitive impairment (CI) in 455 aluminum electrolysis workers from a northern Chinese plant, divided into CI (256) and control (199) groups by MoCA scores. Using inductively coupled plasma mass spectrometry, 11 metals were measured, with analyses via conditional logistic regression,generalized linear models (GLM), Bayesian kernel machine regression (BKMR), and age stratification (40 years). Plasma aluminum (Al), lead (Pb), lithium (Li), manganese, cobalt, and copper were significantly higher in the CI group (all *P* < 0.05), while zinc showed no difference. Single-element analysis found Al, Pb, and Li negatively correlated with MoCA total and subscores (e.g., visuospatial function; *P* < 0.05), and zinc positively correlated with attention (*β* = 1.10, *P* < 0.05). BKMR confirmed metal mixtures above the 25th percentile reduced MoCA scores (*β* = -0.875, 95 % CI: -1.379 to -0.371), with Al, Pb, and Li as key contributors (PIP > 0.6). Subgroup analysis showed Al primarily affected those <40, while Pb had greater impact in those >40. Findings indicate elevated Al, Pb, and Li associate with higher CI risk, metal mixtures synergistically exacerbate impairment, and age modifies these effects, aiding occupational cognitive impairment prevention.

## Highlights

• Workers in aluminum factories with high plasma Al, Pb, and Li levels showed cognitive decline. Zinc may protect cognition.
• Exposure to Al/Pb/Li mixtures above the 25th percentile reduced MoCA scores, showing synergistic neurotoxicity.
• Aluminum caused cognitive impairment in workers <40, while lead dominated in >40, showing age-modified effects.

## 1 Introduction

Aluminum, a key raw material in modern industry, is widely used in electrolysis, smelting, and other industries. Its production process releases a variety of metal elements, including Al, Pb, Mn, and Li [1,2]. The health risks posed by long-term occupational exposure to this metal mixture have become a major concern in the global occupational health field [3]. Workers in aluminum electrolysis plants ingest metal particles through respiratory and dermal contact, leading to accumulation of metal elements in the blood and tissues. Plasma, as a biomarker of metal exposure, directly reflects the level of metal burden in the body [4]. Studies have confirmed that occupational metal exposure is closely associated with neurodegenerative diseases, cardiovascular damage, and metabolic disorders, but the synergistic or antagonistic effects of combined exposure to multiple metals on cognitive function remain poorly characterized [5,6].

The effects of metal elements on the central nervous system are specific and complex [7]. Al, a recognized neurotoxicant, can cross the blood-brain barrier and accumulate in brain regions such as the hippocampus and prefrontal cortex, inducing tau hyperphosphorylation and amyloid deposition, thereby disrupting neuronal synaptic plasticity [8]. Pb exposure interferes with the glutamatergic neurotransmitter system, inhibiting NMDA receptor function and leading to decreased learning and memory abilities [9]. Mn, an essential trace element, can cause basal ganglia damage, manifesting as cognitive flexibility and executive dysfunction, despite excessive exposure [10]. Furthermore, elements such as Li and Co exacerbate neuroinflammation through oxidative stress and mitochondrial dysfunction, while deficiencies in elements such as Zn and Se may weaken the antioxidant defense system, indirectly increasing the risk of cognitive impairment [[11], [12], [13], [14]].

CI, a precursor to dementias such as Alzheimer's disease, has a pathogenesis involving the interaction of multiple factors, including genetics and the environment [15]. Long-term metal exposure has been shown to be a potential risk factor for CI in occupational populations: a cohort study of retired aluminum factory workers showed a negative correlation between plasma Al levels and MoCA scores; another case-control study found that delayed memory ability was significantly reduced in those exposed to Pb [16,17]. However, existing studies have mostly focused on single elements, ignoring the combined effects of metal mixtures in actual exposure scenarios [18]. For example, co-exposure to Al and Pb may amplify neurotoxicity by synergistically activating microglia, while fluctuations in Zn and selenium levels may modulate this process [19,20]. Therefore, analyzing the dose-response relationship between multimetallic exposure profiles and CI is of great significance for clarifying the etiology of occupational cognitive impairment [21].

Based on the above research gaps, this study used Al electrolysis workshop workers as the research subjects. The objectives were: (1) to compare the differences in the levels of 11 metal elements (Al, Pb, Mn, Li, Co, Fe, Zn, Cu, Se, Cr, Cd) in the plasma of the CI group and the control group; (2) to reveal the association between single and combined metal exposure and the risk of CI through single element analysis and Bayesian kernel regression model (BKMR); (3) to explore the specific effects of metal exposure on each sub-item of the MoCA scale (such as visuospatial executive ability and delayed memory) and analyze the modifying effect of factors such as age. By systematically analyzing the relationship between plasma metal element profiles and cognitive function, this study can provide a scientific basis for the early warning and intervention strategy formulation of occupational cognitive impairment.

## 2 Methods

### 2.1 Participants

This study recruited 779 male Al workers from the electrolytic aluminum, alumina, and thermal power plants of a large aluminum smelter in Shanxi Province between July and August 2024. The smelter employs a hybrid production process, a relatively mature process in China, employs approximately 90,000 people, and is the largest alumina producer in Asia. Participants were excluded if they had one or more of the following conditions: 1) members of "other" ethnic groups; 2) experience in Al-related work for less than one year; 3) long-term use of psychotropic medication for more than one month; 4) a parent or immediate family member with a history of neurodegenerative disease; 5) recent major life changes; 6) missing MoCA questionnaire information; 7) extreme uncooperativeness or morbidity during the survey; and 8) participants with aphasia or deafness Fig S1. This study was approved by the Medical Ethics Committee of Shanxi Medical University (No. 2021GLL071), and all participants provided written informed consent.

### 2.2 Basic situation survey

Our research team, comprised of physicians from the Department of Neurology at the First Hospital of Shanxi Medical University and professors and graduate students from the School of Public Health of Shanxi Medical University, all underwent rigorous training. Subsequently, we conducted a large-scale on-site survey at the factory, following an epidemiological survey protocol. All participants completed a questionnaire during a face-to-face interview. The questionnaire collected demographic information, including age, education level, income level, marital status, occupational history, family history, past medical history, and smoking and alcohol use. In this study, smoking was defined as smoking an average of ≥1 cigarette per day for ≥6 months and current smoking. Alcohol consumption was defined as drinking wine, beer, or liquor at least once a week, with a weekly alcohol intake of ≥ 40 g [alcohol intake = volume (ml) × ethanol concentration (%) × 0.8] for ≥ 6 months and current drinking. Physical exercise was defined as exercising at least twice a week, each session for at least 30 min. Income level was defined based on the income of urban and rural residents in Shanxi Province in 2024: ≤ 1562 RMB for low income, 1562 RMB < lower-middle income ≤ 2606 RMB, 2606 RMB < middle income ≤ 3586 RMB, 3586 RMB < upper-middle income ≤ 5977 RMB, and high income >5977 RMB. In addition, we used multiple imputation to fill missing information on covariates for the 455 subjects ultimately included in the study, and selected the set of imputed values with the smallest goodness-of-fit (AIC) value for subsequent analysis Fig S2.

### 2.3 Neuropsychological scale

The Beijing version of the Montreal CognitiveAssessment (MoCA) scale was used to assess the cognitive function of the workers.The total score of the MoCA scale was 30 points, with ≥26 points classified as normal and <26 points classified as CI [22]. The overall cognitive function was evaluated, which included seven cognitive domains: visuospatial and executive function, naming, attention, verbal expression, abstraction, delay, recall and orientation. Recall, Orientation, and 7 cognitive domains. All investigators were trained in a unified manner and applied standardized instructional terms to assess the subjects in a quiet environment.

### 2.4 Plasma metal concentration measurement

2 mL of fasting cubital venous blood was collected from each subject in the morning using sodium heparin anticoagulant tubes. The blood was centrifuged at 1000 rpm for 10 min (centrifugal radius 13.5 cm), and the upper plasma layer was collected for determination of blood metals content. 1.6 mL of 4 % (volume fraction) HNO₃ was added to 400 μL of plasma, and the mixture was digested at room temperature for 24 h. Plasma metal concentrations were determined by inductively coupled plasma mass spectrometry. Standard solutions with varying concentration gradients were prepared from the stock solution, and a standard curve was generated. Samples were tested only after the curve fit exceeded 0.9999. Each sample was tested in duplicate, and after every 10 samples were tested, a standard sample was back-tested for quality control.

### 2.5 Quality control

Before the survey began, investigators received unified training, and the questionnaire used a unified introduction and scoring criteria so that they could master the survey process and requirements. Blood samples were collected, separated, and packaged by professional medical staff, and immediately refrigerated and stored. The same instrument was used to detect blood Al concentration, and unified testing standards were used. All relevant instruments and equipment in the survey were calibrated on time in accordance with metrological certification requirements and guaranteed to be used within the calibration period. Data consistency and logic verification were carried out through double entry.

### 2.6 Statistical analysis

In this study, metal elements with a detection rate greater than 50 % were included in subsequent correlation analyses (Table S1). Data normality was assessed using the Shapiro-Wilk test. Due to the skewed distribution of metal elements, data were log-transformed to base 10. Continuous variables were expressed as mean ± standard deviation or M (*P*_25_, *P*_75_), and categorical variables were expressed as n ( %). Spearman rank correlation analysis was used to analyze correlations between plasma metal elements; a statistical association between two metal elements was considered when *P* < 0.05. Logistic regression models were used to assess the association between individual element exposure and cognitive impairment (CI), and linear trend tests were performed. Generalized linear models (GLMs) were used to assess the association between individual element exposure and MoCA total score, attention and calculation skills, visuospatial executive function, language skills, delayed memory skills, naming skills, abstraction skills, and orientation skills.

Considering the potential for interactions between elements and the potential for nonlinear effects between elements and outcomes, this study used a Bayesian kernel regression model (BKMR) to investigate the associations between exposure to multiple metal elements and MoCA total scores, attention and calculation abilities, visual-spatial executive abilities, language abilities, delayed memory abilities, naming abilities, abstract abilities, and orientation abilities. The theoretical basis of the BKMR model has been elaborated in previous literature [23,24]. In the BKMR model:

$$Y_{i}=h\left(\text{Al},\text{Li},\text{Pb},\text{Mn},\text{Se},\text{Co},\text{Fe},\text{Zn},\text{Cu}\right)+\beta Z_{i}+e_{i}$$

Where Yi represents the continuous outcome of subject i (*i* = 1,…,n), including the total MoCA score and all its sub-items; h() represents an exposure-response function; Al, Li, Pb, Mn, Se, Co, Fe, Zn, and Cu are fitted using log-transformed concentrations; *Z* = Z1,…,Zi are i potential confounders, including age, BMI, marital status, education level, income level, physical exercise, self-reported memory loss, smoking, alcohol consumption, sleep duration, and shift work; *β* represents the effect of the covariate; ei is the residual. In addition, this study calculated the posterior inclusion probability (PIP) value to quantify the relative importance of the influence of metal elements on cognitive scores. The PIP value ranges from 0 to 1, and PIP > 0.5 is used to define elements with significant contributions [25].

Finally, this study's demographic analysis revealed that individuals with CI were older than controls without CI. Given that the age range of the study population was 21–58 years, we conducted subgroup analyses using a cutoff of 40 years to examine the association between exposure to mixed elements and the MoCA total score across different age groups.

All data were analyzed using SPSS 25.0 for Windows (SPSS Inc., Chicago, IL, USA), SAS version 9.4 (SAS Institute Inc., Cary, NC, USA), and R software (version 4.2.2, R Development Core Team). Statistical tests were two-sided, and *P* < 0.05 was considered statistically significant.

## 3 Results

### 3.1 Characteristics of the study population

Based on the MoCA score, this study ultimately included 199 CI cases and 256 non-CI controls. Table 1 describes the basic demographic characteristics of the participants. All subjects were male and of Han ethnicity. Compared with the control group, the CI group was older (*P* < 0.05), more likely to be married (*P* < 0.05), more likely to have less than 9 years of education (*P* < 0.05), more likely to have a low income (*P* < 0.05), and more likely to smoke and work shifts (*P* < 0.05). However, there were no significant differences between the two groups in BMI, physical activity, self-reported memory loss, alcohol consumption, and sleep duration (all *P* > 0.05).

**Table 1:** Demographic characteristics of the two groups of workers.

Variables	Total MoCA scores		^a^*P*-Value
	Control group (*n* = 199)	CI group (*n* = 256)	
Age (years)	36.35±8.47	42.37±8.10	**<0.001**
BMI (kg/m^2^)	24.79±3.72	24.51±3.60	0.406
Marital status, n ( %)			**0.001**
Married	152 (76.4 %)	225 (87.9 %)	
Never married or Divorced	47 (23.6 %)	31 (12.1 %)	
Education level, n ( %)			**<0.001**
< 9 years	43 (21.6 %)	139 (54.3 %)	
≥9 years	156 (78.4 %)	117 (45.7 %)	
Income levels, n ( %)			**<0.001**
Low-income	17 (8.5 %)	51 (19.9 %)	
Lower-middle-income	83 (41.7 %)	132 (51.6 %)	
Middle-income	47 (23.6 %)	38 (14.8 %)	
Upper-middle-income	42 (21.1 %)	25 (9.8 %)	
High-income	10 (5.0 %)	10 (3.9 %)	
Physical exercise, n ( %)			0.827
Yes	93 (46.7 %)	117 (45.7 %)	
No	106 (53.3 %)	139 (54.3 %)	
Self-reported subjective memory decline, n ( %)			0.220
Yes	41 (20.6 %)	77 (30.1 %)	
No	158 (79.4 %)	179 (69.9 %)	
Smoking status, n ( %)			**0.038**
Yes	129 (64.8 %)	189 (73.8 %)	
No	70 (35.2 %)	67 (26.2 %)	
Drinking status, n ( %)			0.659
Yes	47 (23.6 %)	56 (21.9 %)	
No	152 (76.4 %)	200 (78.1 %)	
Sleep duration (h), M (*P*_25_, *P*_75_)	8.0 (7.0, 8.5)	8.0 (7.5, 9.0)	0.088
Shift work, n ( %)			**0.014**
Yes	169 (84.9 %)	236 (92.2 %)	
No	30 (15.1 %)	20 (7.8 %)	

Note: Normally distributed variables were presented as mean±SD. Non-normally distributed variables were presented as median (*P*_25_, *P*_75_). Categorical variables were presented as numbers (percentage). ^a^*P*-Values were derived from Student^’^s *t*-tests or Mann-Whitney U tests for continuous variables according to the data distribution, and chi-square test for the category variables.

### 3.2 Plasma element concentrations

Table 2 summarizes the distribution of nine plasma elements between the two groups. The detection rates of all elements were greater than 80 %. We calculated the M (*P*_25_, *P*_75_) for each element and compared the two groups. Compared with the control group, the CI group had higher concentrations of Al, Pb, Li, Mn, Co, and Cu (all *P* < 0.05). However, there were no statistically significant differences in the concentrations of Se, Fe, and Zn between the two groups (all *P* > 0.05). Figure S3 also shows the Spearman correlations between the elements. Overall, Al was positively correlated with Co, Se, Mn, and Pb (*P* < 0.001), with coefficients ranging from 0.09 to 0.22. Pb was also positively correlated with Co, Se, Mn, and Li (*P* < 0.01), with coefficients ranging from 0.14 to 0.47. Li and Se were both correlated with Cu and Zn, with Li and Zn showing negative correlations (*P* > 0.05). Fe was positively correlated with Zn (*P* < 0.001). The strongest correlation was found between Mn and Co (*R* = 0.57, *P* < 0.001).

**Table 2:** Levels of multiple elements between the two groups (*n* = 455, μg/L).

Compounds	>LOD, *n* ( %)	Median (*P*_25_, *P*_75_)		a*P*-Value
		Control group (≥26)	CI group (<26)	
Al	438 (96.3)	16.23 (8.82 - 36.02)	32.06 (17.17 - 97.05)	**<0.001**
Pb	453 (99.6)	1.15 (0.65 - 1.85)	1.65 (1.15 - 2.55)	**<0.001**
Li	413 (90.8)	5.05 (2.80 - 8.40)	6.80 (4.90 - 11.29)	**<0.001**
Mn	408 (89.7)	1.05 (0.50 - 1.50)	1.30 (0.90 - 2.59)	**<0.001**
Se	453 (99.6)	50.15 (42.05 - 61.30)	53.30 (41.83 - 62.98)	0.196
Co	453 (99.6)	0.05 (0.05 - 0.10)	0.10 (0.05 - 0.25)	**<0.001**
Fe	453 (99.6)	942.75 (738.20 - 1202.40)	893.40 (745.68 - 1130.38)	0.127
Zn	453 (99.6)	936.35 (854.35 - 1014.1)	934.90 (848.45 - 1015.66)	0.627
Cu	453 (99.6)	912.75 (807.80 - 1024.00)	939.68 (857.79 - 1032.16)	**0.020**

Note: LOD: limit of detection.

a *P*-Value were derived from Mann-Whitney U tests.

### 3.3 Correlation between plasma elements and CI risk

We constructed a conditional logistic regression model to explore the association between element levels and CI risk. The concentration of each element was grouped according to tertiles, and the lowest concentration group was used as the reference in the model. As shown in Table 3, the concentrations of Al, Pb, Li, Mn, and Co were significantly associated with CI risk (Model 1). These associations persisted after controlling for potential confounders (Model 2), with all *P* values < 0.05. When controlling for confounding factors, Al, Pb, Li, Mn, and Co were risk factors for CI (Q3 vs. Q1: OR = 4.36, 95 % *CI*: 2.51–7.56; OR = 3.34, 95 % *CI*: 1.94–5.75; OR = 2.61, 95 % *CI*: 1.27–3.62; OR = 3.59, 95 % *CI*: 2.07–6.25; OR = 2.92, 95 % *CI*: 1.77–4.79).

**Table 3:** Odds ratios (ORs) and 95 % confidence intervals (95 % CIs) for the association between each element and CI in the single-factor model.

Elements	OR (95 %CI)			*P*_trend_
	Quartile 1	Quartile 2	Quartile 3	
AI				
Range	≤15.94	∼39.50	>39.50	
CI/Control	57/95	95/56	104/48	
Model 1	1.00 (ref)	2.83 (1.78, 4.50)***	3.61 (2.25, 5.80)***	**<0.001**
Model 2	1.00 (ref)	3.79 (2.20, 6.51)***	4.36 (2.51, 7.56)***	**<0.001**
Pb				
Range	≤1.05	∼1.90	>1.90	
CI/Control	59/93	92/63	105/43	
Model 1	1.00 (ref)	2.30 (1.46, 3.64)***	3.85 (2.34, 6.23)***	**<0.001**
Model 2	1.00 (ref)	2.28 (1.35, 3.83)**	3.34 (1.94, 5.75)***	**<0.001**
Li				
Range	≤4.85	∼8.40	>8.40	
CI/Control	63/91	91/59	102/49	
Model 1	1.00 (ref)	2.23 (1.41, 3.53)***	3.01 (1.88, 4.80)***	**<0.001**
Model 2	1.00 (ref)	2.17 (1.29, 3.62)**	2.16 (1.27, 3.65)**	**0.013**
Mn				
Range	≤0.90	∼1.65	>1.65	
CI/Control	71/85	80/71	105/43	
Model 1	1.00 (ref)	1.35 (0.86, 2.11)	2.92 (1.82, 4.70)***	**<0.001**
Model 2	1.00 (ref)	1.39 (0.83, 2.31)	3.59 (2.07, 6.25)***	**<0.001**
Se				
Range	≤46.10	∼58.90	>58.90	
CI/Control	80/72	89/66	87/61	
Model 1	1.00 (ref)	1.21 (0.77, 1.90)	1.28 (0.81, 2.03)	0.298
Model 2	1.00 (ref)	1.26 (0.76, 2.11)	1.20 (0.72, 2.01)	0.349
Co				
Range	≤0.05	∼0.10	>0.10	
CI/Control	119/131	31/24	106/44	
Model 1	1.00 (ref)	1.42 (0.79, 2.56)	2.65 (1.73, 4.08)***	**<0.001**
Model 2	1.00 (ref)	1.24 (0.63, 2.47)	2.92 (1.77, 4.79)***	**<0.001**
Fe				
Range	≤798.65	∼1056.35	>1056.35	
CI/Control	85/67	94/58	77/74	
Model 1	1.00 (ref)	1.28 (0.81, 2.02)	0.82 (0.52, 1.29)	0.292
Model 2	1.00 (ref)	1.42 (0.84, 2.39)	1.03 (0.61, 1.72)	0.594
Zn				
Range	≤884.55	∼992.90	>992.90	
CI/Control	89/63	85/67	82/69	
Model 1	1.00 (ref)	0.90 (0.57, 1.41)	0.84 (0.53, 1.33)	0.455
Model 2	1.00 (ref)	0.08 (0.64, 1.81)	1.07 (0.64, 1.81)	0.793
Cu				
Range	≤871.05	∼989.15	>989.15	
CI/Control	71/81	96/56	89/62	
Model 1	1.00 (ref)	1.96 (1.24, 3.09)**	1.64 (1.04, 2.58)*	0.051
Model 2	1.00 (ref)	1.40 (0.83, 2.36)	1.11 (0.65, 1.88)	0.797

Note: Model 1: No covariates were adjusted. Model 2: Adjusted for age, BMI, marital status, education, income level, physical activity, self-reported memory decline, smoking and drinking status, sleep duration, and shift work. * indicates *P* < 0.05; ** indicates *P* < 0.01; *** indicates *P* < 0.001.

### 3.4 Correlation between plasma elements and MoCA total score and its sub-items

Generalized linear regression models were used to analyze the correlations between log-transformed element levels and the MoCA total score and its subscores. The results showed (Table 4) that plasma Al levels were significantly negatively correlated with the MoCA total score (*P* < 0.001), attention and calculation ability (*P* < 0.05), visuospatial executive function (*P* < 0.05), language ability (*P* < 0.01), and delayed memory ability (*P* < 0.01). Plasma Pb levels were significantly negatively correlated with the MoCA total score (*P* < 0.01), delayed memory ability (*P* < 0.01), and naming ability (*P* < 0.05). Plasma Li levels were significantly negatively correlated with the MoCA total score (*P* < 0.05) and visuospatial executive function (*P* < 0.001). Plasma Zn levels were significantly positively correlated with attention and calculation ability (*P* < 0.05). Specifically, controlling for confounding factors, each logarithmic increase in plasma Al concentration was associated with decreases of 1.09 points in the MoCA total score, 0.16 points in the Attention and Calculation score, 0.18 points in the Visuospatial Executive Skills score, 0.23 points in the Language Skills score, and 0.35 points in the Delayed Memory Skills score, respectively. Each logarithmic increase in plasma Pb concentration was associated with decreases of 1.40 points in the MoCA total score, 0.66 points in the Delayed Memory Skills score, and 0.13 points in the Naming Skills score, respectively. Each logarithmic increase in plasma Li concentration was associated with decreases of 0.55 points in the MoCA total score and 0.24 points in the Visuospatial Executive Skills score, respectively. Each logarithmic increase in plasma Zn concentration was associated with an increase of 1.10 points in the Attention and Calculation Skills score. In this analysis, no correlations were found between the elements and abstraction and orientation skills, nor were there any correlations between Mn, Se, Co, Fe, and Cu and cognitive scores.

**Table 4:** Correlation analysis^a^ of multiple metal elements with the MoCA total score and its sub-items.

Compounds	*β* (95 %CI)							
	Total MoCA Score	Attention and Calculation Ability	Visuospatial/Executive Ability	Language Ability	Delayed Recall Ability	Naming Ability	Abstract Ability	Orientation Ability
Al^b^	−1.09 (−1.65, −0.53)***	−0.16 (−0.29, −0.03)*	−0.18 (−0.34, −0.02)*	−0.23 (−0.37, −0.09)**	−0.35 (−0.60, −0.10)**	−0.03 (−0.10, 0.05)	−0.11 (−0.24, 0.01)	−0.03 (−0.15, 0.09)
Pb^b^	−1.40 (−2.33, −0.46)**	−0.22 (−0.44, 0.002)	−0.13 (−0.4, 0.13)	−0.10 (−0.33, 0.13)	−0.66 (−1.08, −0.24)**	−0.13 (−0.25, −0.01)*	−0.17 (−0.38, 0.03)	0.02 (−0.18, 0.22)
Li^b^	−0.55 (−1.06, −0.05)*	−0.24 (−0.36, −0.12)***	−0.04 (−0.18, 0.11)	0.01 (−0.12, 0.13)	−0.16 (−0.39, 0.07)	−0.05 (−0.11, 0.02)	−0.02 (−0.13, 0.09)	−0.05 (−0.16, 0.06)
Mn^b^	0.67 (−0.31, 1.65)	0.22 (−0.02, 0.45)	0.02 (−0.26, 0.3)	0.22 (−0.02, 0.46)	0.14 (−0.30, 0.58)	0.02 (−0.10, 0.15)	0.03 (−0.19, 0.24)	0.03 (−0.18, 0.24)
Se^b^	−0.41 (−2.36, 1.53)	−0.22 (−0.68, 0.24)	−0.18 (−0.73, 0.38)	−0.02 (−0.51, 0.46)	0.26 (−0.62, 1.14)	0.05 (−0.20, 0.30)	−0.22 (−0.64, 0.21)	−0.09 (−0.51, 0.33)
Co^b^	−0.24 (−0.94, 0.45)	−0.03 (−0.20, 0.13)	0.05 (−0.15, 0.25)	−0.12 (−0.29, 0.06)	−0.07 (−0.38, 0.25)	0.06 (−0.03, 0.15)	−0.12 (−0.27, 0.04)	−0.02 (−0.17, 0.13)
Fe^b^	−0.07 (−2.36, 2.22)	−0.33 (−0.87, 0.22)	0.02 (−0.63, 0.67)	0.37 (−0.20, 0.94)	−0.06 (−1.08, 0.97)	−0.01 (−0.30, 0.29)	−0.06 (−0.56, 0.44)	−0.02 (−0.51, 0.48)
Zn^b^	2.94 (−1.28, 7.16)	1.10 (0.10, 2.11)*	0.78 (−0.42, 1.98)	−0.23 (−1.29, 0.82)	0.24 (−1.65, 2.14)	0.37 (−0.17, 0.91)	0.57 (−0.36, 1.49)	0.11 (−0.80, 1.01)
Cu^b^	−2.31 (−5.78, 1.16)	−0.57 (−1.40, 0.25)	−0.82 (−1.81, 0.17)	−0.17 (−1.04, 0.69)	−0.10 (−1.66, 1.47)	−0.39 (−0.84, 0.05)	−0.34 (−1.11, 0.42)	0.08 (−0.66, 0.83)

Note: a: Generalized linear models were employed for the analysis. b: Plasma metal elements concentrations were log–transformed. The model was adjusted for age, BMI, marital status, education, income level, physical activity, self-reported memory decline, smoking and drinking status, sleep duration, and shift work. * indicates *P* < 0.05; ** indicates *P* < 0.01; *** indicates *P* < 0.001.

### 3.5 Bayesian kernel machine regression (BKMR) analysis

We applied the BKMR model to fit the effects of mixed exposure to multiple elements on cognitive scores. First, we calculated the probability of each element (PIP) (Table S2). This probability can be considered a measure of the element's importance: values closer to 1 indicate greater importance, while values closer to 0 indicate less importance. Specifically, Al contributed 0.841, 0.799, 0.868, and 0.680 to the MoCA total score, delayed memory score, language ability score, and abstract ability score, respectively. Pb contributed 0.689, 0.952, and 0.755 to the MoCA total score, delayed memory score, and abstract ability score, respectively. Li contributed significantly to the attention and calculation ability scores (0.995).

**Fig. 1:**
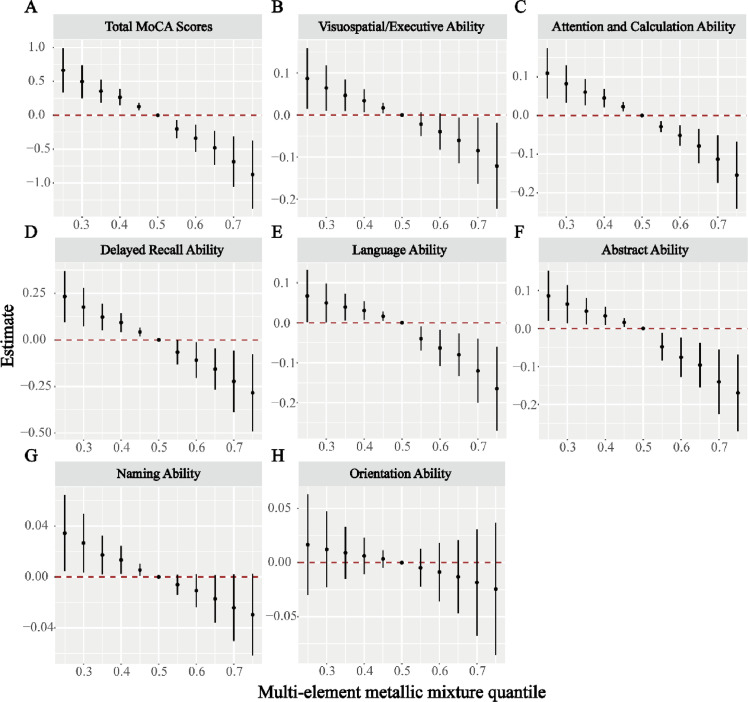
Compared with exposure at the median, the overall effect of the metal mixture when each element was fixed at different percentiles. (A) Total MoCA scores, (B) Visuospatial/Executive Ability, (C) Attention and Calculation Ability, (D) Delayed Recall Ability, (E) Language Ability, (F) Abstract Ability, (G) Naming Ability, (H) Orientation-Ability.

We then fitted the cumulative effects of the element mixture relative to its median concentration when the element mixture was uniformly fixed at different percentiles (from *P*_25_ to *P*_75_, with a step size of 5 %). The results showed that when the element mixture exposure level was above the 25th percentile or higher, its joint effect on the MoCA total score was statistically significant (Fig. 1A). Specifically, when the element mixture concentration was adjusted to the 75th percentile, each percentile increase in the mixture concentration was associated with a decrease in the MoCA total score of 0.875 points (95 % *CI*: 0.371, 1.379). Similarly, when the element mixture exposure level was above the 65th percentile or higher, its joint effect on visuospatial executive function was statistically significant (Fig. 1B). When the element mixture exposure level was above the 25th percentile or higher, its joint effects on attention and calculation ability (Fig. 1C), delayed memory ability (Fig. 1D), language (Fig. 1E), and abstract ability (Fig. 1F) were statistically significant. Although naming (Fig. 1G) and orientation abilities (Fig. 1H) also showed a downward trend with increasing concentrations of element mixtures, no statistical differences were found.

**Fig. 2:**
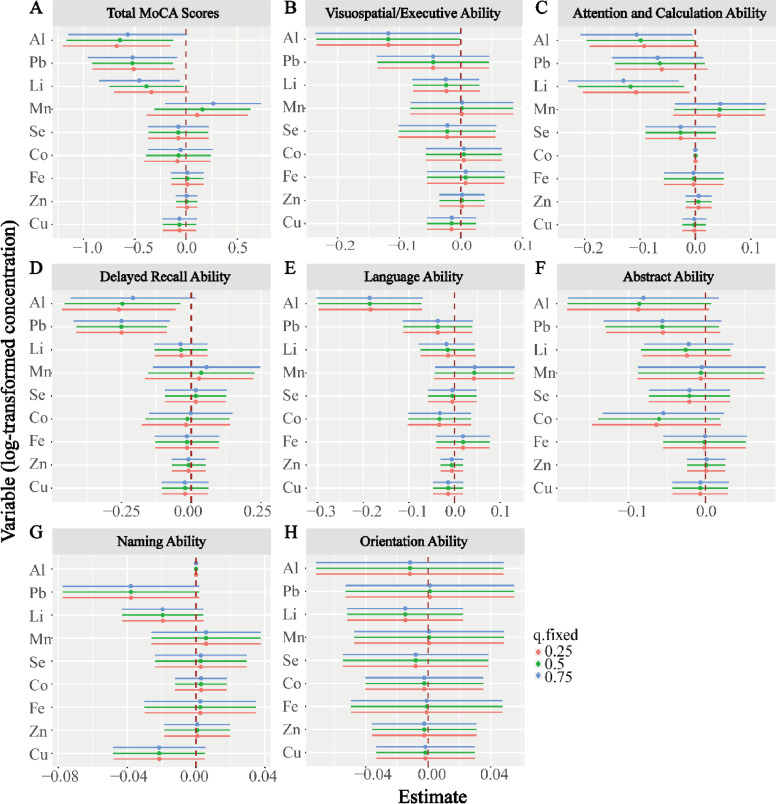
The effect of a single element when all other elements are fixed at their 25th, 50th, and 75th percentiles. (A) Total MoCA scores, (B) Visuospatial/Executive Ability, (C) Attention and Calculation Ability, (D) Delayed Recall Ability, (E) Language Ability, (F) Delayed Recall Ability, (G) Naming Ability, (H) Orientation Ability.

Next, we assessed the cumulative effects of individual element exposures. Specifically, we assessed the impact of individual element exposures on cognitive scores when the other elements were fixed at their 25th, 50th, or 75th quantiles. For example, the results showed (Fig. 2A) that exposure to Al, Pb, or Li significantly contributed to the reduction in the MoCA total score. Specifically, when the other elements were fixed at their 25th quantile, each logarithmic unit increase in Al, Pb, or Li concentration was associated with a decrease in the MoCA total score of 0.676 (95 % CI: 0.149, 1.203), 0.511 (95 % CI: 0.111, 0.911), or 0.338 (95 % CI: 0.027, 0.703), respectively. When the other elements were fixed at their 50th percentile, each logarithmic unit increase in the concentration of Al, Pb, or Li was associated with a decrease in the MoCA total score by 0.645 (95 % CI: 0.124, 1.166), 0.523 (95 % CI: 0.127, 0.919), or 0.386 (95 % CI: 0.025, 0.747), respectively. When the other elements were fixed at their 75th percentile, each logarithmic unit increase in the concentration of Pb or Li was associated with a decrease in the MoCA total score by 0.522 (95 % CI: 0.087, 0.957) or 0.455 (95 % CI: 0.063, 0.847), respectively. Similarly, the results showed that Al exposure primarily affected visuospatial executive function (Fig. 2B), attention and calculation abilities (Fig. 2C), delayed memory (Fig. 2D), and language abilities (Fig. 2E) in occupational Al workers. Li mainly affected the attention and calculation abilities of workers exposed to Al (Fig. 2C), while Pb mainly affected the delayed memory abilities of workers exposed to Al (Fig. 2D).

**Fig. 3:**
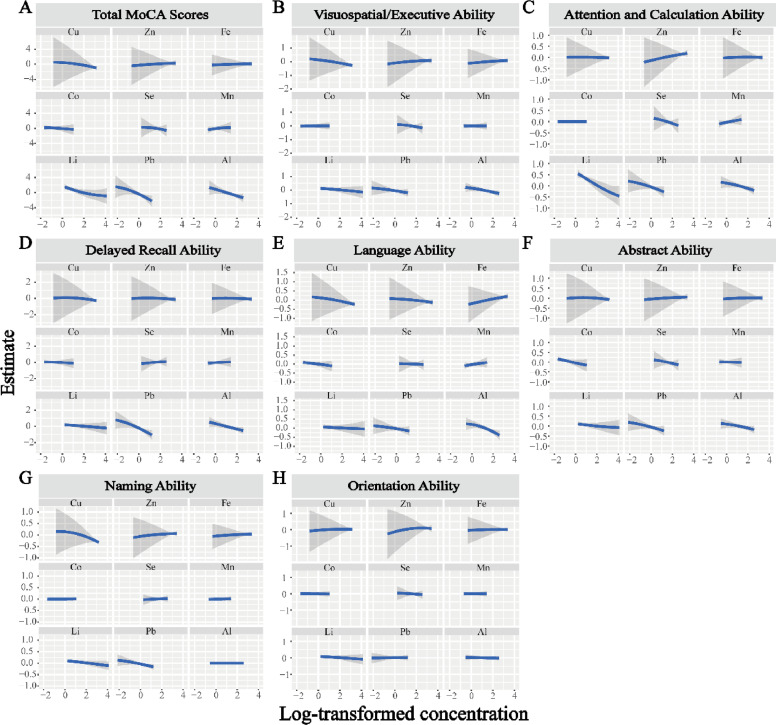
The univariate exposure–response function and its 95 % confidence band, with all other elements held at their 50th. (A) Total MoCA scores, (B) Visuospatial/Executive Ability, (C) Attention and Calculation Ability, (D) Delayed Recall Ability, (E) Languagel Ability, (F) Abstract Ability, (G) Naming Ability, (H) Orientation Ability.

We then fitted an exposure-response function (95 % *CI*) between each element and the cognitive score, with the other elements fixed at their 50th percentile. As shown in Fig. 3, the exposure-response curves for Al, Pb, or Li and the MoCA total score were consistent with the positive trend found in the conditional logistic regression results, showing a linear decreasing trend. Similarly, the exposure-response curves for Al and visuospatial executive function (Fig. 3B), attention and calculation ability (Fig. 3C), delayed memory ability (Fig. 3D), and language ability (Fig. 3E) all showed a linear decreasing trend. Furthermore, the Li element and attention and calculation ability (Fig. 3C) and Pb and delayed memory ability (Fig. 3D) also showed a linear decreasing trend.

**Fig. 4:**
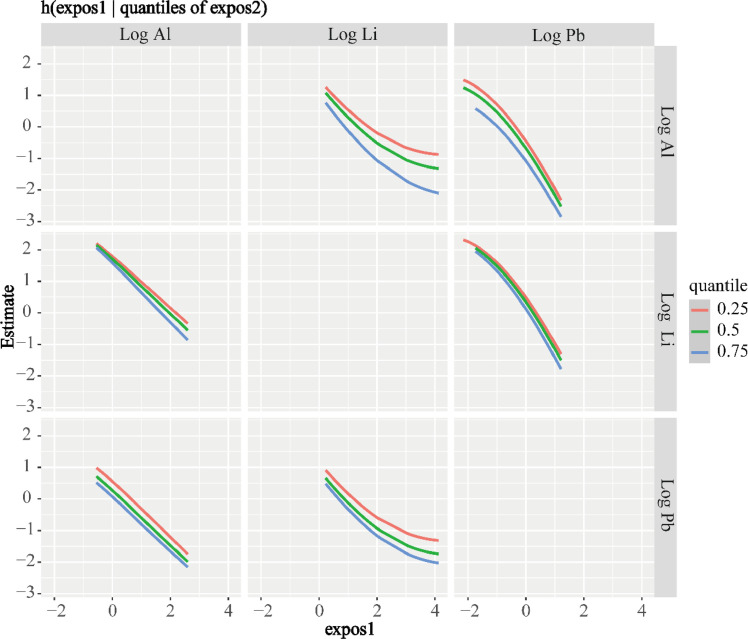
Bivariate exposure–response function analysis. “expos1” denotes the three elements displayed along the horizontal axis—namely, log-transformed Al, log-transformed Li, and log-transformed Pb.

Finally, combining the previous results, we fitted bivariate exposure-response results for Al, Pb, and Li exposures to the MoCA total score. As shown in Fig. 4, each row represents "expos 2," fixed at its 25th, 50th, and 75th percentiles, respectively; each column represents "expos 1" for the element under investigation, with the remaining elements fixed at the median. Overall, the trends of the bivariate curves are generally consistent with those of the univariate curves. However, in this study, the bivariate curves for Al, Pb, and Li did not cross, indicating the absence of an interaction effect.

### 3.6 Subgroup analysis

**Fig. 5:**
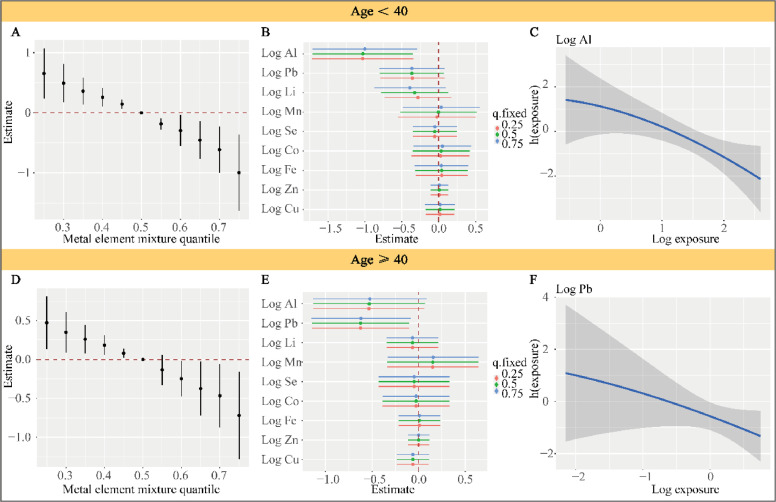
Subgroup analysis stratified by age. Note: The outcome was the total MoCA score.

After stratification by age, the joint effects of elemental exposure and MoCA total score remained statistically significant (Fig. 5A and 5D), indicating that this effect was unaffected by age. However, among individuals under 40 years of age, Al was the element that contributed most significantly to this joint effect (Fig. 5B). In contrast, among individuals over 40 years of age, Pb was the most important element contributing to the joint effect (Fig. 5E). Furthermore, the univariate exposure-response functions for Al (Fig. 5C) and Li (Fig. 5F) with MoCA total score showed a linear decreasing trend, consistent with the unstratified results.

## 4 Discussions

This study systematically analyzed the distribution of 11 metal elements in the plasma of Al factory workers and their association with CI, revealing the potential impact of occupational metal exposure on human health. The results showed that compared with the control group, the plasma concentrations of Al, Pb, Li, Mn, Co, and Cu in the CI group were significantly increased, while Zn levels were significantly decreased. This finding provides new insights into the neurotoxic mechanisms of metal mixtures.

The cumulative effect of Al was most prominent in this study. Plasma Al concentrations in the CI group were nearly twofold higher than in the control group, consistent with the findings of Shang et al., suggesting that occupational exposure to Al may be a key driver of cognitive impairment [26]. Al can cross the blood-brain barrier and accumulate in the hippocampus and prefrontal cortex, interfering with neuronal synaptic plasticity by inducing tau protein hyperphosphorylation and β-amyloid protein (Aβ) aggregation [8]. In addition, Al can also aggravate oxidative stress and damage mitochondrial function by inhibiting glutathione peroxidase activity [27]. Pb is a classic neurotoxin. In this study, elevated concentrations of Pb were associated with an increased risk of CI (OR=3.34, 95 % CI:1.94–5.75).This is consistent with the dose-response relationship between Pb exposure and cardiovascular disease risk found by Hu et al., suggesting that Pb may affect health through multi-system toxicity [28]. The mechanisms may involve: a. interference with blood-brain barrier integrity, increasing the release of neuroinflammatory factors (such as IL-6 and TNF-α). b. inhibition of δ-aminolevulinic acid dehydratase activity, leading to neurotransmitter metabolism disorders [29]. Li levels were abnormally elevated in this study and significantly correlated with decreased visuospatial performance (β = −0.24, *P* < 0.001). This finding may be related to the high-dose nature of occupational exposure.This finding may be related to the high-dose nature of occupational exposure [30]. High concentrations of Li can disrupt neuronal calcium homeostasis and induce cell apoptosis by inhibiting the overactivation of glycogen synthase kinase-3β (GSK-3β) [31]. Another key finding was elevated levels of Mn and Co. Mn is a key cofactor in the mitochondrial respiratory chain, but excessive exposure can induce oxidative stress by inducing microglial activation [32]. Co, on the other hand, may exacerbate neuronal damage by interfering with vitamin B12 metabolism and DNA repair mechanisms [33]. The synergistic increase of the two in the CI group suggests that metal mixtures may amplify neurotoxicity through additive effects [34]. Decreased Zn levels are of particular significance. As a regulator of brain-derived neurotrophic factor (BDNF), Zn deficiency impairs synaptic formation and antioxidant defenses [35]. In this study, the positive correlation between Zn and attention and computational abilities (*β* = 1.10, *P* < 0.05) supports its role in cognitive protection, which may be related to zinc's regulatory function on NMDA receptors [36].

Bayesian kernel regression (BKMR) analysis showed that when the metal mixture was ≥25th percentile, the MoCA total score decreased by 0.875 points (95 % CI: 0.371–1.379), among which the joint effect of Al, Pb, and Li contributed the most (PIP>0.6).This synergistic effect may occur through the following mechanisms: a. Activation of the oxidative stress network: Al and Pb synergistically induce NADPH oxidase activity, increasing reactive oxygen species (ROS) production; reduced zinc further impairs the scavenging capacity of the glutathione system, forming an "oxidative stress amplification loop" [37,38]. b. Metal transporter competition: Al and Fe share the transferrin receptor (TfR), potentially reducing cellular iron uptake through competitive binding, exacerbating iron deficiency-related energy metabolism disorders in the brain [39]. c. Neuroinflammatory cascade: Co and Mn can promote the release of proinflammatory cytokines by activating the NF-κB pathway, while Li may exacerbate neuroinflammation by interfering with microglial polarization (M1/M2 imbalance) [40,41]. It is worth noting that this study found complex correlations between metals: Al was positively correlated with Co, Se, Mn, and Pb (*r* = 0.09–0.22, *P* < 0.001), while Pb had the strongest correlation with Co (*r* = 0.47, *P* < 0.001), suggesting that these elements may enter the human body through common exposure pathways (such as respiratory inhalation) and have mutual influences during metabolism in the body [42,43].

The occupational exposure of Al factory workers has the following characteristics: a. High exposure dose: The plasma Al concentration of electrolytic Al workers is much higher than that of the normal population [26]; b. Long exposure time: The average length of service is ≥10 years, and the cumulative exposure is significantly higher than that of the general population [44]; c. Mixed exposure: In addition to Al, it is also accompanied by chemicals such as fluoride and sulfur dioxide, which may aggravate toxicity through "metal-chemical" interactions [45]. This study found that workers in the CI group were older (42.37 ± 8.10), had a higher smoking rate (73.8 %), and were more likely to work shifts (92.2 %). These factors may synergize with metal exposure: increased age reduces metal metabolism capacity, smoking can exacerbate oxidative stress through the synergistic effects of elements such as Cd, and circadian rhythm disruption caused by shift work may further weaken neuroprotective mechanisms (such as decreased melatonin secretion) [46,47]. Subgroup analysis found that age was an important effect modifier: in people <40 years old, Al was the main contributor to cognitive impairment (PIP=0.72), while the effect of Pb was more prominent in people >40 years old (PIP=0.63), which may be related to differences in metal metabolism capacity and blood-brain barrier integrity in different age groups [48].

This study is the first to systematically reveal the differential distribution of 11 metals in the plasma of Al factory workers and their associations with cognitive function, revealing the synergistic neurotoxicity of Al, Pb, and Li. The BKMR model overcomes the limitations of traditional single-element analysis, confirming the central role of Al and Pb in cognitive impairment and providing new evidence for understanding the combined toxicity of metal mixtures. However, limitations include: a. The cross-sectional design precludes the determination of causal relationships. b. Time-effect data on metal exposure are still insufficient. Future research should employ cohort designs, combining metal speciation analysis (such as different chemical forms of aluminum) with high-throughput transcriptomics, and integrating humoral and imaging biomarkers to elucidate the chain reaction mechanism of "exposure-metabolism-cognitive impairment".

## 5 Conclusions

This study clarified the association between occupational metal exposure and cognitive impairment in Al factory workers. Results showed that plasma levels of Al, Pb, and Li were significantly elevated in the CI group, while Zn levels were decreased. Both single-element and mixed exposures were associated with cognitive decline. These findings provide a scientific basis for establishing occupational exposure limits and early intervention strategies.

## Ethical approval

This study was approved by the Medical Ethics Committee of Shanxi Medical University (No. 2021GLL071), and all subjects signed informed consent.

## Funding

This work was supported by the Shanxi Higher Education Science and Technology Innovation Project 2023L104.

## Declaration of generative AI and AI-assisted technologies in the writing process

Generative AI and AI-assisted technologies were not used during the preparation of this manuscript.

## Data availability

The research data contains sensitive information. Please contact the corresponding author to obtain it.

## CRediT authorship contribution statement

**Xin Guo:** Writing – original draft, Investigation, Funding acquisition. **Fangyu Gao:** Formal analysis, Data curation. **Mujia Li:** Methodology, Investigation. **Baolong Pan:** Data curation, Conceptualization. **Feng Gao:** Supervision. **Shanshan Wang:** Investigation. **Jingsi Zhang:** Investigation. **Xiaoting Lu:** Investigation. **Jing Song:** Investigation. **Linping Wang:** Investigation. **Huifang Zhang:** Investigation. **Qiao Niu:** Writing – review & editing, Supervision, Conceptualization.

## Declaration of competing interest

All authors have no competing interests or personal relationships.

## Appendix Supplementary materials

Image, application 1
