# Supplementary material for: Association between plasma metal element profiles and cognitive impairment in occupationally aluminum-exposed workers at a large aluminum plant in northern China
Source: J Prev Alzheimers Dis. 2026 Jan 1;13(3):100470. doi: 10.1016/j.tjpad.2025.100470 (PMC12988366; doi:10.1016/j.tjpad.2025.100470)
Supplement: Supplementary file 1 [file mmc1.docx]

**Association between plasma metal element profiles and mild cognitive impairment in occupationally aluminum-exposed workers at a large aluminum plant in northern China**

**Author names**

Xin Guo^1, 2, 3^, Fangyu Gao^2, 3^, Mujia Li^2^, Baolong Pan^3^, Feng Gao^3^, Shanshan Wang^2^, Jingsi Zhang^2^, Xiaoting Lu^2^, Jing Song^2^, Linping Wang^2^, Huifang Zhang^2^, Qiao Niu^1, 3,^ *

***Corresponding author and email address**

Qiao Niu, School of Public Health, Shanxi Medical University, Taiyuan, Shanxi 030001, China. E-mail: [NiuQiao55@163.com](mailto:NiuQiao55@163.com)


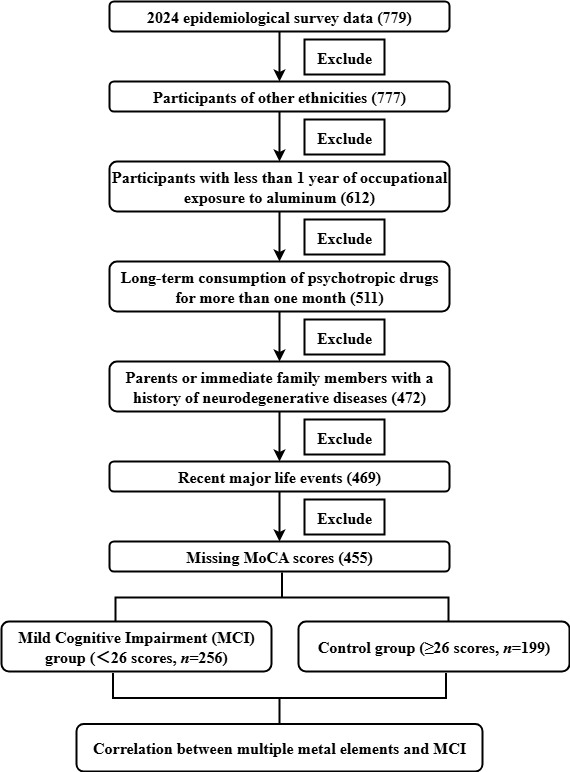


Fig S1. Inclusion and exclusion criteria


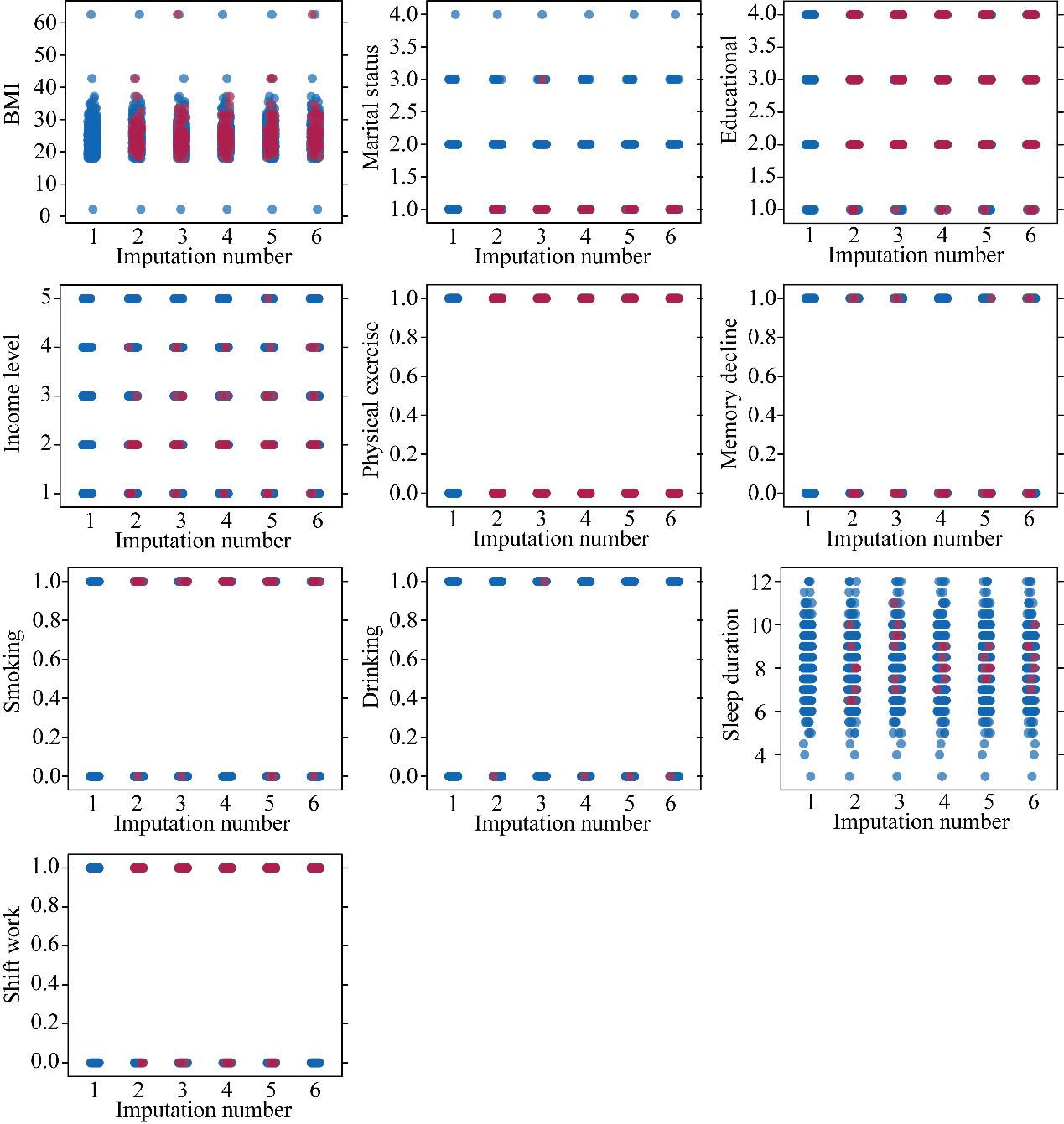


|  | AIC |
| --- | --- |
| 1 | 843.9 |
| 2 | 849.8 |
| 3 | 836.9 |
| 4 | 841.3 |
| 5 | 842.6 |

Fig S2. covariate imputation

Table S1. Detection of multiple metal elements in plasma of occupational Al-exposed workers (*n*=779)

| Elements | LOD/(μg/L) | Detection rate/% |
| --- | --- | --- |
| Li | 2.35 | 83.70 |
| Cr | 0.39 | 25.03 |
| Mn | 0.29 | 85.24 |
| Fe | 0.66 | 99.10 |
| Se | 2.48 | 99.10 |
| Co | 0.03 | 99.23 |
| Cu | 0.21 | 99.10 |
| Zn | 0.30 | 99.10 |
| Pb | 0.01 | 99.10 |
| Cd | 0.10 | 46.85 |
| Al | 0.39 | 93.20 |


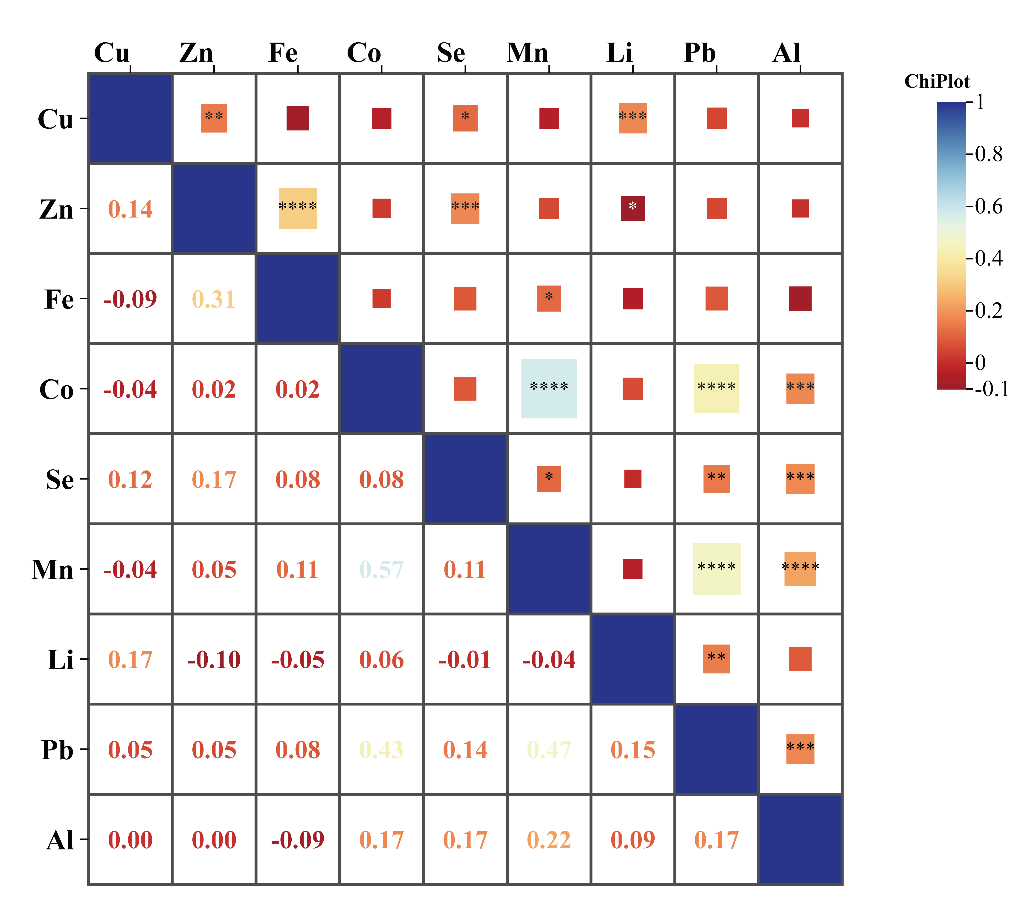


Fig S3. The Spearman correlation heatmap of plasma elements’ levels among the overall participants (*n*=455). * indicates *P*<0.05; ** indicates *P*<0.01; *** indicates *P* < 0.001; **** indicates *P* < 0.0001.

Table S2. Posterior Inclusion Probability (PIP) derived from the BKMR fit

| Elements | PIP^b^ | | | | | | | |
| --- | --- | --- | --- | --- | --- | --- | --- | --- |
|  | Total MoCA score | Visuospatial/  Executive Ability | Attention and Calculation Ability | Delayed Recall Ability | Language Ability | Abstract Ability | Naming Ability | Orientation Ability |
| Al^a^ | **0.841** | 0.279 | 0.203 | **0.799** | **0.868** | **0.680** | 0.000 | 0.010 |
| Pb^a^ | **0.689** | 0.072 | 0.222 | **0.952** | 0.118 | **0.755** | 0.144 | 0.030 |
| Li^a^ | 0.442 | 0.039 | **0.995** | 0.203 | 0.165 | 0.479 | 0.050 | 0.021 |
| Mn^a^ | 0.098 | 0.051 | 0.063 | 0.310 | 0.086 | 0.474 | 0.022 | 0.015 |
| Se^a^ | 0.183 | 0.267 | 0.224 | 0.385 | 0.121 | **0.628** | 0.080 | 0.081 |
| Co^a^ | 0.053 | 0.028 | 0.000 | 0.359 | 0.100 | **0.817** | 0.003 | 0.008 |
| Fe^a^ | 0.021 | 0.116 | 0.066 | 0.247 | 0.128 | **0.528** | 0.033 | 0.052 |
| Zn^a^ | 0.045 | 0.140 | 0.080 | 0.359 | 0.124 | **0.556** | 0.150 | 0.244 |
| Cu^a^ | 0.139 | 0.166 | 0.044 | 0.347 | 0.126 | **0.552** | 0.315 | 0.112 |

Note: a: Plasma metal elements concentrations were log–transformed. b indicates that the multiple elements were included in the model across 5,000 iterations of the MCMC sampler.
